# HOSPITALIZATION-ASSOCIATED FUNCTIONAL DECLINE IN OLDER ADULTS: CLASSIFYING THE RISK USING THE “TIMED UP AND GO” TEST

**DOI:** 10.1093/geroni/igae098.0426

**Published:** 2024-12-31

**Authors:** Roy Tzemah-Shahar, Orly Gatenio-Hefling, Kfir Asraf, Omer Dilian, Efrat Gil, Maayan Agmon

**Affiliations:** University of Haifa, Haifa, HaZafon, Israel; University of Haifa, Haifa, Hefa, Israel; Yezreel Valley College, Afula, HaZafon, Israel; University of Haifa, Haifa, Hefa, Israel; HaEmek Medical Center, Afula, HaZafon, Israel; University of Haifa, University of Haifa, HaZafon, Israel

## Abstract

Hospitalization Associated Functional Decline (HAFD) in older adults has been recognized as a major phenomenon with crucial consequences such as slower recovery increased morbidity, and higher mortality; it is linked with reduced mobility levels during hospitalization. To date, there is no objective test that can directly estimate personal risk for HAFD. The aim of this study was to examine the contribution of the Timed Up and Go (TUG) test in predicting HAFD among older adults. We used a cross-sectional study design; a total of 310 older adults (age≥65) hospitalized between December 2018 and August 2020 were included; the Modified Barthel Index was used to assess HAFD; it was administered at admission to evaluate patients’ independence in activities of daily living two weeks prior hospitalization, and at discharge. The TUG test was performed on admission; a cut-score of 12 seconds was used to predict functional decline, while accounting for demographics, length of hospitalization, comorbidity burden (Charlson’s comorbidity index), and Adult Lifestyles and Function Interview (ALFI-MMSE). Participants able to complete the TUG test in less than 12 seconds showed no significant change in the Modified Barthel Index; TUG test completion in 12 seconds and over or inability to complete it was associated with significant reduction in function, defined by a reduction of three points or more in the Modified Barthel Index (a reduction of 4 and 8.8 points respectively). The risk for HAFD is currently underestimated in clinical settings, the TUG test could be used to identify those at risk of HAFD.

